# Study on friction and wear properties of nickel-based alloys under different working conditions

**DOI:** 10.1016/j.heliyon.2025.e41797

**Published:** 2025-01-15

**Authors:** Ming Li, Yongjun Bai, Yi Ou, Xiaoyu Ren, Chaoqun Qian

**Affiliations:** a Nanjing University of Science and Technology, Nanjing, 210094, China; b Shanghai Nuclear Engineering Research and Design Institute Co., Ltd, Shanghai, 200233, China

**Keywords:** Friction and wear, S-N curve, Nickel-based alloys, Wear rate, Coefficient of friction

## Abstract

This study systematically analyzes the friction and wear properties, as well as the fatigue life characteristics, of high-hardness nickel-based alloys under specialized working conditions. The primary contribution lies in elucidating the frictional behavior and wear mechanisms of these alloys under varying rotational speeds, loads, and lubrication environments, while establishing S-N curves specifically tailored to nickel-based alloys. These findings provide theoretical support for optimizing their engineering applications in extreme environments. Friction and wear tests are conducted using a Bruker UMT Tribolab wear testing machine to examine surface wear morphology. A white-light interferometer is employed to measure wear profiles and calculate wear rates, while a fatigue testing machine collects stress-life data to model S-N curves. Experimental results show that increasing rotational speed slightly raises the average coefficient of friction, with adhesion on the material surface positively correlated to speed. The wear rate initially decreases and then increases with higher rotational speeds. Under increasing load, the fluctuation amplitude of the friction coefficient diminishes and stabilizes, accompanied by a gradual decline in its average value. The furrow phenomenon on the surface correlates positively with load, and while the wear rate decreases with increasing load, the rate of decrease slows over time. The formation of a lubricating film contributes to the uniform distribution of the load, and a positive correlation is observed between the wear rate and the coefficient of friction. Fitting results of the S-N curve reveal that the fatigue life of nickel-based alloys adheres closely to an exponential function relationship. This study offers critical theoretical insights to support the design and application of nickel-based alloys in extreme conditions, such as those encountered in nuclear power systems.

## Highlights

• The study investigated the friction and wear properties, as well as fatigue life characteristics, of nickel-based alloys under varying rotational speeds, loads, and lubrication conditions.
• The friction coefficient of the nickel-based alloy increased progressively with rotational speed, indicating a significant effect of rotational speed on frictional heat generation.
• The wear rate initially decreased and then increased with rotational speed, suggesting that elevated temperatures at high speeds lead to re-adhesion of material.
• As the load increased, the fluctuation of the friction coefficient gradually decreased and stabilized, while the wear rate decreased with the increasing load.
• An S-N curve model for the nickel-based alloy was established, revealing the material's fatigue life characteristics, which follow an exponential function relationship.

## 1 Introduction

Drive mechanisms operating in water must maintain stable performance under extreme conditions, making material selection critical. Nickel-based alloys, renowned for their exceptional high-temperature strength, corrosion resistance, and oxidation resistance, are particularly well-suited for such environments [[1], [2], [3]]. In contrast, conventional martensitic alloys often fail to deliver adequate performance under these demanding conditions. Consequently, investigating the friction and wear behavior, along with the fatigue S-N curves of nickel-based alloys in aqueous environments, is essential to ensuring the reliability and durability of drive mechanisms in practical applications.

Feng [4] carried out friction and wear tests on nickel-based alloys by using a ball disc testing machine at different temperatures and studied the friction-induced structures of wear surfaces at different temperatures. KS Rama [5] studied the friction and wear properties of nickel-based alloy coatings under dry friction conditions at room temperature. The research showed that as the loads increased, the wear mechanism of nickel-based alloy coatings changed from slight micro-cutting wear and fatigue wear to abrasive wear and micro-crack wear. Zx [6] explored the mechanisms by which different grain sizes affect friction force, wear depth, and the coefficient of friction. Xu Xiangyang [7] studied the fretting wear behavior of nickel-based alloys. Chen Baiming [8] used a needle-disc friction and wear testing machine to evaluate the friction and wear properties of nickel-based alloy materials. Jie Li [9] studied the effects of grain size and heat treatment hardness on the friction and wear properties of nickel-based alloys, finding that nickel-based alloys with small grains and high hardness exhibit better wear resistance. Tan Helong [10] used a friction and wear testing machine with controllable roll-slip ratio to study the friction and wear properties of GCR15 bearing steel under different abrasive particle sizes. J liu [11] studied the friction and wear properties of 18Cr-8Ni austenitic stainless steel under dry sliding at room temperature. Yuan be [12] studied the microstructure, friction and wear properties of SiC_p_/ZL101 composites after casting. Jiao lei [13] studied the microstructure and high-temperature friction and wear properties of (AlB2 + Al2O3)/A356 composite materials.

Park Jae Phil [14] established a probabilistic fatigue life model for Ni-based alloys using the fatigue data from the NUREG/CR-6909 report and the statistical analysis of the new fatigue data for the 52M/152 and 82/182 alloys. Zhugang [15] conducted the axial load fatigue test of nickel-based alloys to establish a new fatigue life model based on the internal microstructure-induced cracking mechanism. Vanhari Afrooz kazemi [16] derived new constraints based on the fitting of the S-N fatigue curve and the mathematical curve of the material, which decreased the complexity of the nonlinear optimization in the Sendeckyj wear model. Liuyang Feng [17] proposed a new and effective method, which was used to estimate the S-N fatigue life curve of high-strength steels under low-temperature and low-cycle environments.

Existing research on the tribological properties and lifespan models of nickel-based alloy self-lubricating materials and coatings has achieved notable progress. However, most studies primarily evaluate material properties under standard operating conditions, with limited focus on the overall aging properties of nickel-based alloy materials under specialized working conditions. This study addresses this gap by conducting friction and wear tests on nickel-based alloys used in nuclear power drive devices. The tests are performed under varying loads (5, 10, and 15 N), rotational speeds (100, 200, and 300 RPM), and lubrication environments (water, air, and lubricating oil) to investigate the tribological behavior of these materials and calculate their wear rates. Additionally, fatigue tests are conducted to establish an S-N curve model for nickel-based alloys, providing a comprehensive understanding of their mechanical performance under extreme conditions.

## 2 Test materials and equipment

### 2.1 Chemical composition of the material

The nickel-based alloy bars are fabricated using chromium (Cr) and nickel (Ni) as the primary matrix elements, with small amounts of other elements added. These elements are thoroughly and uniformly mixed, followed by solution treatment and forging to produce the final alloy bars. The specific chemical composition of the material, along with the proportion of each element, is detailed in Table 1. The bars undergo aging treatment at 740 °C for 5 h to achieve a hardness of 53 HRC, and their chemical composition is also provided in Table 1. The specimens used in the wear and fatigue tests are machined from these nickel-based alloy bars, with a hardness of 53 HRC, using hard turning. Their dimensions are $\emptyset_{m}52\times 6.6mm$ and $\emptyset_{p}60\times 5mm$. For the wear tests, the grinding balls are made from a nickel-based alloy with the same composition and a hardness of 58 HRC, and their diameter is ∅7.144mm.

**Table 1:** Chemical composition of nickel-based alloys.

elements	melting analysis	product analysis
C	≤0.03	≤0.03
Mn	≤1.00	≤1.00
P	≤0.02	≤0.02
S	≤0.005	≤0.005
Al	2.60–3.80	≤2.50–3.80
Cr	38.00–43.00	37.8–43.00
Ni	allowance	allowance
Co	≤0.05	≤0.05

### 2.2 Friction and wear test

**Fig. 1:**
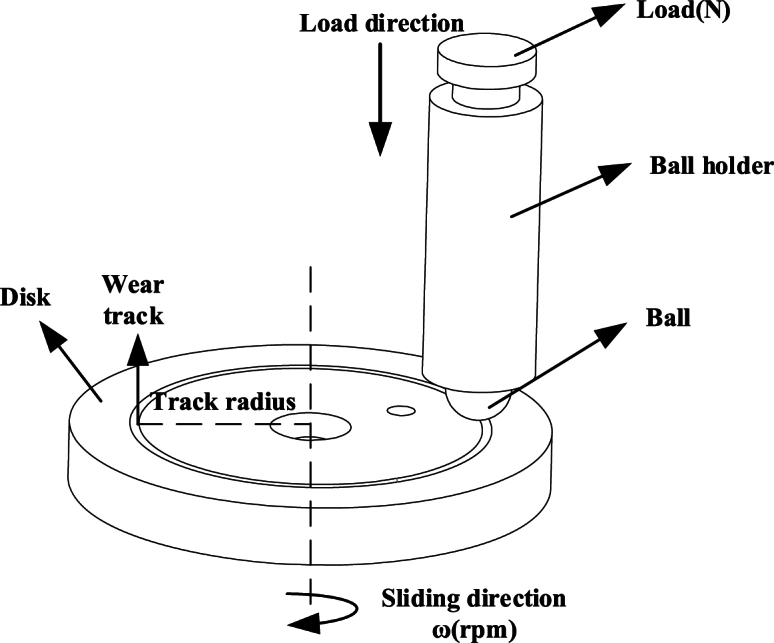
Schematic diagram of the working principle of rotary wear.

Ball-disc friction and wear tests are conducted using the rotating module of the Bruker UMT Tribolab friction and wear testing machine. In this setup, the ball is fixed as the upper specimen on a loading device equipped with sensors to measure friction in the horizontal direction and load in the vertical direction. The vertical load is continuously adjustable to ensure that the contact force between the ball and the specimen remains at the set value throughout the test (see Fig. 1).

### 2.3 Fatigue test

**Fig. 2:**
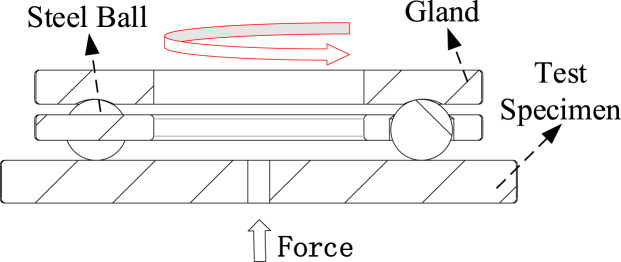
Schematic diagram of the wear fatigue testing machine.

The working principle of the testing machine is as follows: The gland is interference-fitted to the rotating shaft, and a servo motor drives the shaft via a belt, causing the gland to rotate synchronously. During contact with the steel ball, the gland applies a certain amount of extrusion force, which, in turn, causes the ball to roll under frictional force, completing the fatigue wear test. The entire test container is supported by the steel ball, ensuring higher pair contact between the ball and the container. This arrangement prevents the test specimen from experiencing excessive localized loads, as illustrated in Fig. 2.

## 3 Test method

### 3.1 Friction and wear test plan

**Fig. 3:**
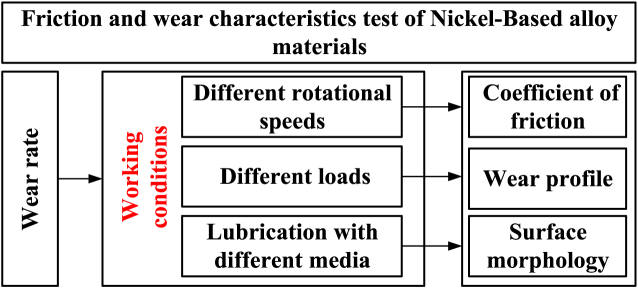
Flow chart of friction and wear test of nickel-based alloy materials.

Friction and wear tests on nickel-based alloy materials are conducted under various working conditions to investigate their friction and wear characteristics. The specific experimental workflow is illustrated in Fig. 3.

### 3.2 Friction and wear test cut-off conditions

**Fig. 4:**
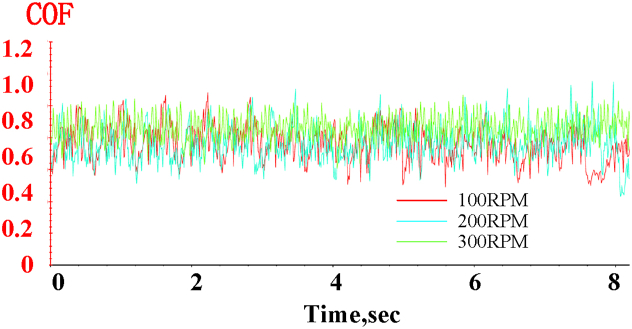
Segmented coefficient of friction.

In the friction and wear experiments, a pre-wear test protocol is designed to accurately measure the coefficient of friction of nickel-based alloys and assess any potential influence of the testing machine's start-stop cycle on the measurements. Given the short run-in period of nickel-based alloys, the tests are conducted under a load of 50 N, a rotational speed of 100 RPM, and in an air-lubricated environment, divided into four consecutive 5-min intervals. The experimental results show that during the first 5-min interval, the coefficient of friction increases rapidly at the beginning and stabilizes within approximately 5 s. In the subsequent three intervals, the fluctuation amplitude of the coefficient remains consistent, as shown in Fig. 4. This suggests that the initial wear phase of the specimen surface has a significant effect on the coefficient of friction. However, once the specimen reaches the steady-state wear phase, the fluctuations of the coefficient stabilize. Based on these observations, to improve experimental efficiency and ensure data reliability, the duration of subsequent friction and wear tests is standardized to 5 min.

### 3.3 Wear rate calculation

According to the Archard wear theory, the wear rate is [18]:

(1) $$K=\frac{V}{Qvt}$$

In the formula: V is the wear volume; Q is the test load; v is the relative sliding linear velocity between the grinding two objects; and t is the grinding time.

## 4 Trial analysis

### 4.1 Effect of rotational speed on friction and wear characteristics

(1) Effect of rotational speed on the friction factor

**Fig. 5:**
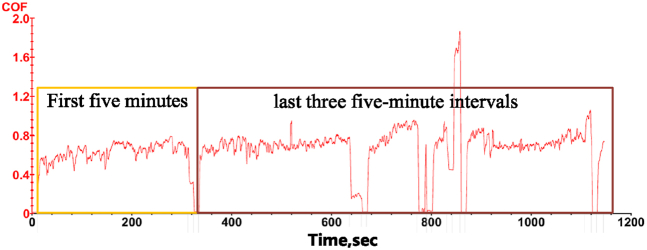
Variation of the friction coefficient over time at rotational speeds of 100 RPM, 200 RPM, and 300 RPM under a 50N load and air lubrication conditions.

The variation in the friction factor of nickel-based materials over time at different rotational speeds, under a 50 N load and air medium conditions, is shown in Fig. 5. As observed from the figure:

The friction factor of the specimens fluctuates within a certain range over time at different rotational speeds, but the amplitude of these fluctuations does not vary significantly with increasing speed.

The average friction factor exhibits a slight upward trend as rotational speed increases. At 100 RPM, the average friction factor is 0.7664; at 200 RPM, it rises to 0.7985, a 4 % increase compared to 100 RPM; and at 300 RPM, it reaches 0.8739, approximately 14 % higher than at 100 RPM. This phenomenon may be attributed to high-frequency contact between the grinding ball and the specimen, which increases local temperature and generates frictional heat [19]. This heat softens the material and leads to adhesion, thus raising the friction factor.

This analysis suggests that the effect of rotational speed on the average friction coefficient is more significant than its impact on the fluctuations in the friction factor.

(2) Effect of rotational speed on wear surfaces and wear profiles

**Fig. 6:**
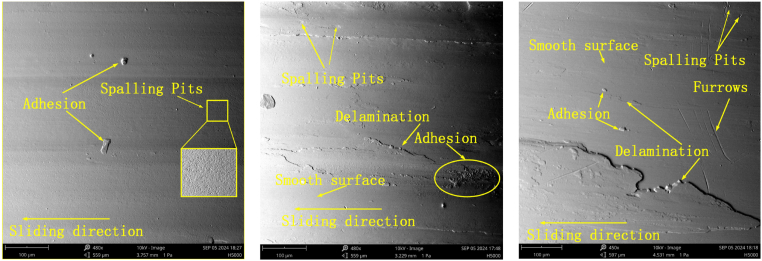
Wear morphology of the specimen at rotational speeds of 100 RPM, 200 RPM, and 300 RPM under a 50N load and air lubrication conditions.

**Fig. 7:**
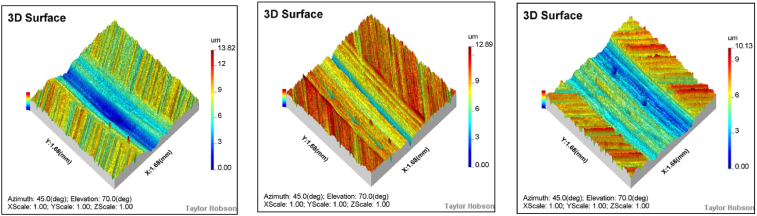
Three-dimensional wear profiles of the specimen at rotational speeds of 100 RPM, 200 RPM, and 300 RPM under a 50N load and air lubrication conditions.

**Fig. 8:**
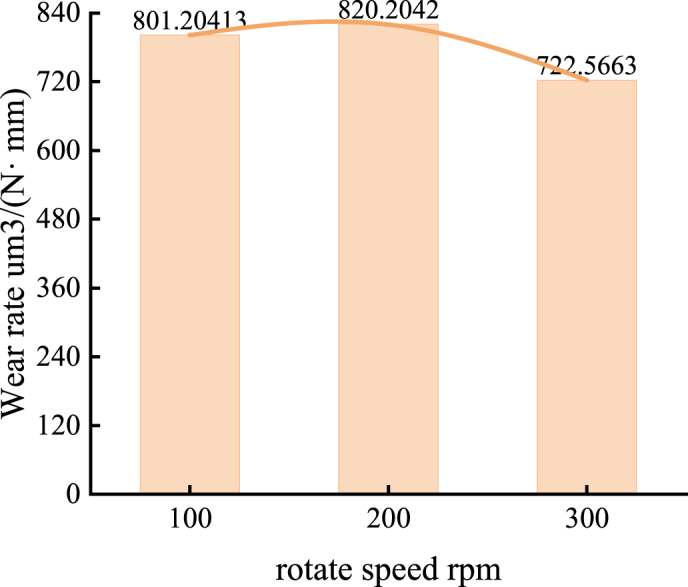
Wear rate of the specimen at rotational speeds of 100 RPM, 200 RPM, and 300 RPM under a 50N load and air lubrication conditions.

Under a 50 N load and air lubrication conditions, the wear surfaces and profiles of the specimens at different rotational speeds are shown in Fig. 6, Fig. 7. Observed phenomena, such as adhesion, spalling pits, furrows, and material delamination, indicate that the specimens have experienced adhesive wear, fatigue wear, and abrasive wear. As shown in Fig. 6(a–c), the number of spalling pits decreases progressively, while the adhesion phenomenon intensifies as rotational speed increases. This can be attributed to the heat generated by the high rotational speeds, which induces intense molecular motion and weakens the stability of van der Waals bonds. As a result, the nickel-based alloys become more prone to delaminating from the nickel matrix in powder form. These fine powders fill the spalling pits and, under external pressure, combine with the pits, leading to material adhesion and the formation of a relatively smooth surface. Concurrently, some particles adhere to the specimen surface, resulting in a bulge phenomenon (see Fig. 8).

The wear width increases with rotational speed, while the wear depth decreases as speed rises. At 100 RPM, the wear depth is 13.8 μm and the width is 730 μm; at 200 RPM, the wear depth is 12.9 μm and the width is 743 μm; and at 300 RPM, the wear depth is 10.1 μm and the width is 979 μm. Notably, the wear depth does not increase proportionally with rotational speed, which can be attributed to the abrasive debris generated during the wear process being influenced by the furrow effect, making it difficult to detach from the contact area. Additionally, as rotational speed increases, the temperature between the steel ball and the specimen surface rises. Under these high-temperature conditions, abrasive debris may undergo sintering or reattachment, forming a protective layer that inhibits further material removal.

Additionally, a significant disparity exists between the wear width and depth, which can be attributed to material loss between the grinding ball and the specimen. This leads to a transition from the original point-to-surface contact to surface-to-surface contact.

As rotational speed increases, the wear rate initially decreases and then rises. At 100 RPM, the wear rate is highest, reaching 801.2 μm³/(N·mm). At 200 RPM, the wear rate increases slightly to 820.2 μm³/(N·mm), but then decreases to 722.6 μm³/(N·mm) at 300 RPM, which is 0.902 times the rate at 100 RPM. This trend can be attributed to the rise in temperature at the friction interface, which significantly promotes material reattachment, leading to a lower wear rate at 300 RPM compared to other rotational speeds.

### 4.2 Effect of load on friction and wear characteristics

(1) Effect of load on coefficient of friction

**Fig. 9:**
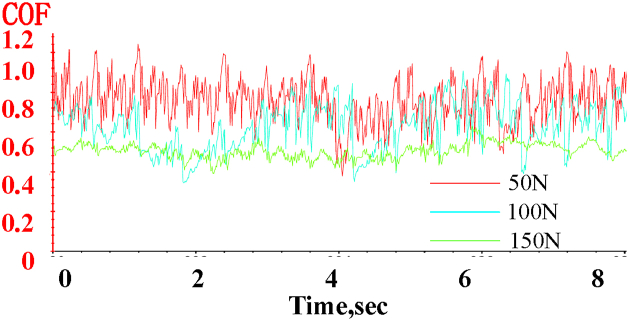
Variation of the friction coefficient over time under different loads (50N, 100N, and 150N) at 100 RPM and air lubrication conditions.

Fig. 9 shows the variation of the friction factor of the specimen over time under different loads at 100 RPM and air lubrication conditions.

The friction factor of the specimen fluctuates within a certain range over time under different loads, with the fluctuation amplitude gradually stabilizing as the load increases. Under a 50 N load, the friction factor exhibits poor stability, with fluctuations ranging from 0.62 to 0.98. However, under a 150 N load, the stability improves significantly, with the fluctuation range narrowing to 0.42 to 0.58, a reduction of approximately 32 % compared to the 50 N load.

The average friction factor decreases as the load increases. At a load of 50 N, the average friction factor is 0.7664. As the load rises to 100 N, the average value decreases to 0.6592, approximately 14 % lower than at 50 N. With a further increase to 150 N, the average friction factor drops to 0.4874, about 36 % lower than at 50 N.

This phenomenon can be attributed to the increased load, which facilitates the extrusion of carbon elements (C) from the matrix gaps, while the relative sliding motion promotes the oxidation of carbides. The oxidation reaction forms a protective film on the specimen's surface. Additionally, under higher load conditions, the increased heat generated by the contact between the steel ball and the specimen further accelerates the oxidation process. As a result, the protective oxide layer becomes more pronounced under higher loading, effectively reducing the coefficient of friction [[20], [21], [22], [23]].

(2) Effect of load on the wear surface and wear profile

**Fig. 10:**
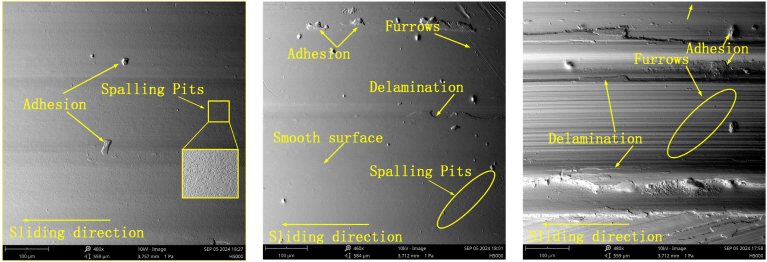
Wear morphology of the specimen under different loads (50N, 100N, and 150N) at 100 RPM and air lubrication conditions.

**Fig. 11:**
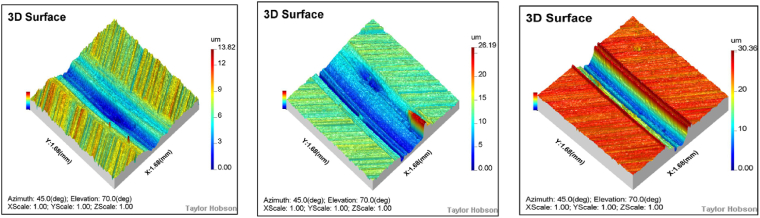
Three-dimensional wear profiles of the specimen under different loads (50N, 100N, and 150N) at 100 RPM and air lubrication conditions.

A detailed observation and analysis of the wear behavior of the specimen under different loads are conducted at 100 RPM and air lubrication conditions. As shown in Fig. 10, Fig. 11, the wear morphology and profile of the specimen exhibit significant changes with increasing load. Under a 50 N load, the specimen surface primarily shows small areas of spalling pits, with a relatively light wear degree and a wear depth of only 13.8 μm. However, when the load increases to 100 N, adhesion and delamination appear on the surface, along with a small number of furrows. The wear degree also increases, with the wear depth reaching 20.2 μm, approximately 1.89 times that observed at 50 N. Further increasing the load to 150 N leads to extensive furrow formation, significantly exacerbating the wear degree. The wear depth reaches 30.4 μm, which is 2.2 times greater than that under the 50 N load.

It is important to note that the hardness of the disc specimen is significantly lower than that of the grinding ball, which is the primary reason for the increased wear degree as the load rises. As the load increases, the pressure on the specimen surface also increases, making it easier for furrows to form in the material. The wear profile analysis reveals a clear proportional relationship between wear depth and load—specifically, as the load increases, so does the wear depth. This finding underscores the substantial effect of load on wear degree and further supports the conclusion that the lower hardness of the disc specimen, compared to the grinding ball, is a key factor contributing to the formation of furrows.

**Fig. 12:**
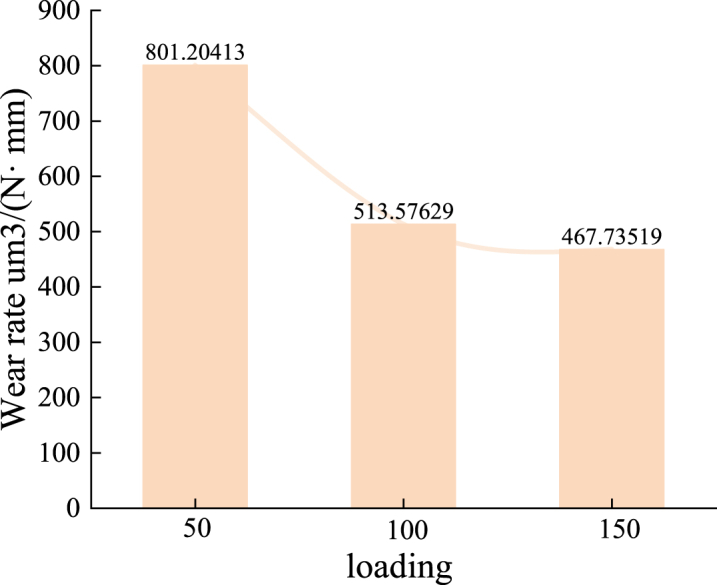
Wear rate of the specimen under different loads (50N, 100N, and 150N) at 100 RPM and air lubrication conditions.

The wear rate decreases with increasing load, although the rate of decrease gradually slows, as shown in Fig. 12. At a load of 50 N, the wear rate of the nickel-based alloy materials is 801.2 μm³/(N·mm). When the load increases to 100 N, the wear rate drops to 513.6 μm³/(N·mm), approximately 64 % of the wear rate at 50 N. As the load increases further to 150 N, the wear rate decreases to 467.7 μm³/(N·mm), about 58 % of the wear rate at 50 N.

### 4.3 Effect of medium lubrication on friction and wear characteristics

(1) Effect of medium lubrication on coefficient of friction

**Fig. 13:**
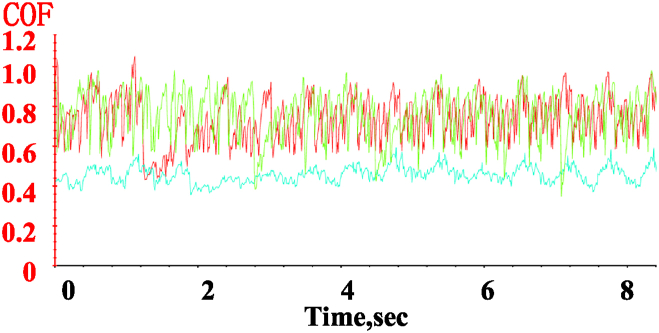
Variation of the friction coefficient over time under different lubricating media (air, water, and oil) at 50N load and 100 RPM.

The variation in the friction factor of the specimen over time under different medium conditions at 100 RPM and a 50 N load is shown in Fig. 13. Under different medium conditions, the friction factor fluctuates within a certain range, with both the amplitude and average value being significantly influenced by the type of medium. In the air medium, the average value and fluctuation amplitude of the friction factor are the largest, with the fluctuation ranging from 0.59 to 0.93 and an average value of 0.7664. In the water medium, the fluctuation range is from 0.59 to 0.92, with an average value of 0.754, approximately 98 % of the value observed in the air medium. Under oil lubrication, the friction factor fluctuates between 0.38 and 0.52, with an average value of 0.407, about 53 % of the value observed in the air medium. This analysis highlights the significant impact of good lubrication on frictional properties.

(2) Effect of medium lubrication on wear surfaces and wear profiles

**Fig. 14:**
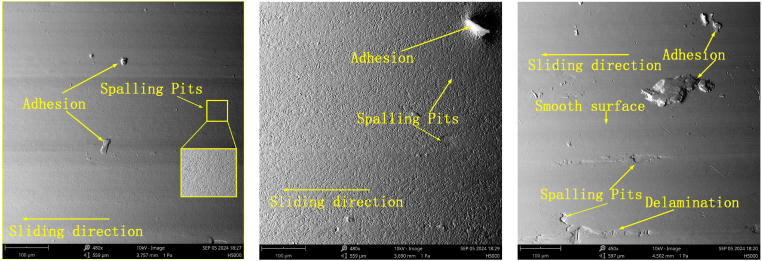
Wear morphology of the specimen under different lubricating media (air, water, and oil) at 50N load and 100 RPM.

**Fig. 15:**
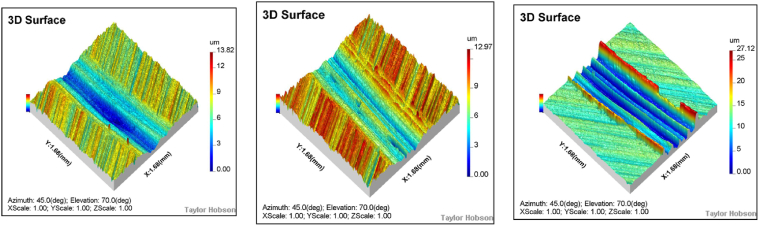
Three-dimensional wear profiles of the specimen under different lubricating media (air, water, and oil) at 50N load and 100 RPM.

**Fig. 16:**
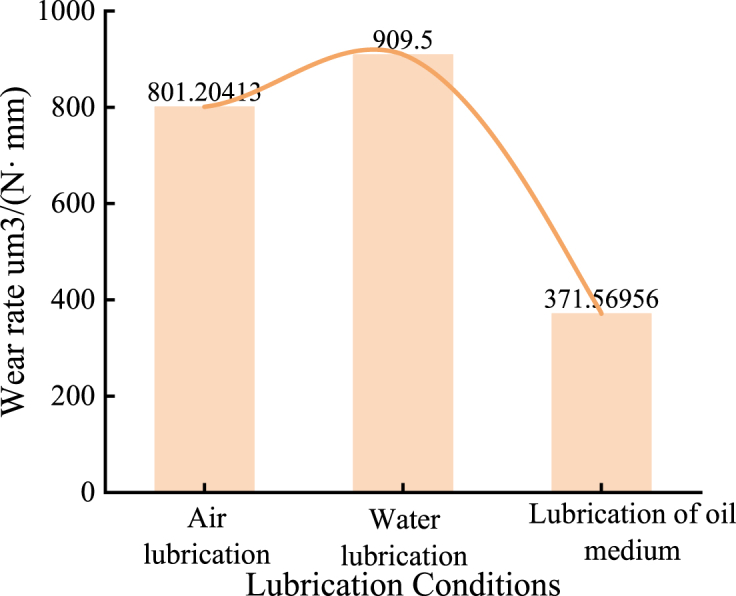
Wear morphology and wear rate of the specimen at rotational speeds of 100 RPM, 200 RPM, and 300 RPM under a 50N load and air lubrication conditions.

The wear morphology and profiles of the specimen under 100 RPM and 50 N load conditions are shown in Fig. 14, Fig. 15. The wear characteristics on the specimen surface differ significantly under the three medium conditions: air, water, and oil. Under water lubrication, the specimen exhibits more pronounced spalling pits compared to the air medium. This is because the fine material particles generated during wear are carried away by the water flow, reducing the significant material adsorption in the spalling pits caused by fatigue. In contrast, under oil lubrication, the wear surface is smoother due to the formation of an oil film, which helps distribute the Hertzian contact stress more evenly and reduces localized wear. Material reattachment is observed under all three medium conditions, primarily because the grinding ball and the specimen material are the same, resulting in good compatibility between the two (see Fig. 16).

Under air lubrication conditions, the wear scar depth is 13.8 μm and the width is 730.8 μm. Under water lubrication, the wear scar depth decreases to 12.9 μm, and the width increases slightly to 735.8 μm, approximately 93 % and 101 % of the wear scar width and depth under air lubrication conditions, respectively. In contrast, under oil lubrication conditions, the wear scar depth decreases to 8 μm, and the width reduces to 575.0 μm, approximately 38 % and 78 % of the wear scar width and depth under air lubrication conditions, respectively.

The wear rate is significantly influenced by the type of lubricating medium. Under air lubrication conditions, the wear rate of the nickel-based alloy material is 801.2 μm³/(N·mm). Under water lubrication, the wear rate increases to 909.5μm³/(N·mm), approximately 1.14 times higher than that observed under air lubrication. In contrast, under oil lubrication conditions, the wear rate decreases to 371.6 μm³/(N·mm), about 46 % of the wear rate under air lubrication. These findings indicate a positive correlation between the wear rate and the friction coefficient.

### 4.4 S-N curve of nickel-based alloy materials

**Fig. 17:**
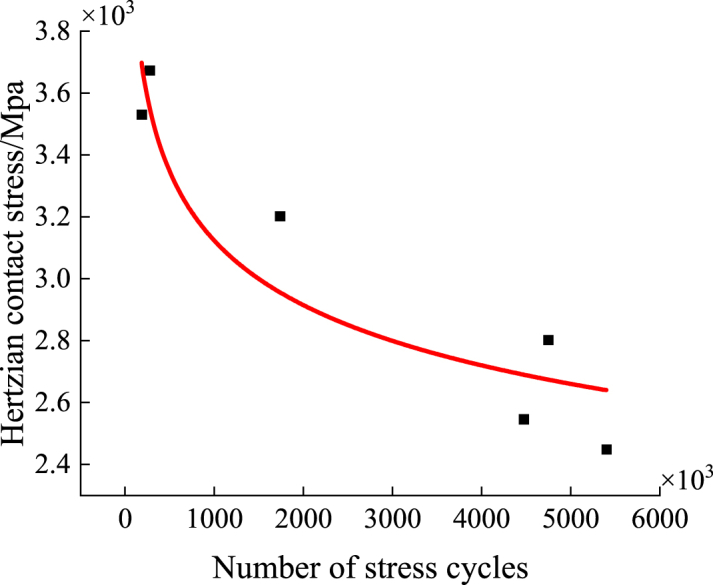
S–N curve of nickel-based alloy materials.

The test is conducted with incremental loading of 500 N, and the test is terminated when the vibration induced by the steel ball rolling over the specimen reaches 3G. This level of vibration is similar to the impact produced when the steel ball strikes the specimen at three times the velocity of free fall. The fitted curve of the point set after the test approximates a power function, as shown in Fig. 17. Consequently, the power function model from the fatigue S-N curve for metallic materials is adopted as the fatigue curve model for this material [24]. The approximate fitting formula is as follows:

(2) $$\sigma^{m}N_{f}=C$$

In the formula, m and C are parameters related to the material stress ratio, loading method, and so on; $N_{f}$ is the fatigue life under the current load; σ is the stress level.

The final fitted function is:

(3) $$\sigma^{7.27}N=2.39\times 10^{31}$$

## 5 Conclusions of the test

(1) The rotational speed has a minor impact on the fluctuation value of the friction factor; however, the average friction factor exhibits a slight increase with rising rotational speed. This is primarily due to the elevated local temperature at the specimen surface, which softens the material and increases the phenomenon of adhesion. Despite this, the impact of rotational speed on the wear rate is negligible.
(2) As the load increases, both the fluctuation and average values of the friction factor decrease. While the load has a relatively small effect on the fluctuation value, its impact on the average friction factor is more pronounced. The increased load intensifies the furrow phenomenon on the specimen surface, and the wear rate decreases, though the rate of decrease gradually slows down.
(3) The formation of a lubricating film aids in the uniform distribution of load and provides effective protection, which reduces the wear rate. The friction coefficient is positively correlated with the wear rate, meaning that reducing the friction coefficient is an effective strategy to lower the wear rate.
(4) The S-N curve of nickel-based alloys approaches a power function form. The specific fatigue life curve can be expressed by the following formula: $\sigma^{7.27}N=2.39\times 10^{31}$.

## CRediT authorship contribution statement

**Ming Li:** Writing – original draft. **Yongjun Bai:** Writing – review & editing. **Yi Ou:** Conceptualization. **Xiaoyu Ren:** Conceptualization. **Chaoqun Qian:** Conceptualization.

## Declaration of competing interest

The authors declare that they have no known competing financial interests or personal relationships that could have appeared to influence the work reported in this paper.
